# The Accuracy of the Electrocardiogram during Exercise Stress Test Based on Heart Size

**DOI:** 10.1371/journal.pone.0023044

**Published:** 2011-08-17

**Authors:** Jason C. Siegler, Shafiq Rehman, Geetha P. Bhumireddy, Raushan Abdula, Igor Klem, Sorin J. Brener, Leonard Lee, Christopher C. Dunbar, Barry Saul, Terrence J. Sacchi, John F. Heitner

**Affiliations:** 1 Department of Sport, Health & Exercise Science, University of Hull, Hull, United Kingdom; 2 Division Cardiology, New York Methodist Hospital, Brooklyn, New York, United States of America; 3 Brooklyn College of the City University of New York, New York, New York, United States of America; 4 Division of Cardiothoracic Surgery, New York Methodist Hospital, Brooklyn, New York, United States of America; 5 Division of Cardiology, Duke Medical Center, Durham, North Carolina, United States of America; Pennington Biomedical Research Center, United States of America

## Abstract

**Background:**

Multiple studies have shown that the exercise electrocardiogram (ECG) is less accurate for predicting ischemia, especially in women, and there is additional evidence to suggest that heart size may affect its diagnostic accuracy.

**Hypothesis:**

The purpose of this investigation was to assess the diagnostic accuracy of the exercise ECG based on heart size.

**Methods:**

We evaluated 1,011 consecutive patients who were referred for an exercise nuclear stress test. Patients were divided into two groups: small heart size defined as left ventricular end diastolic volume (LVEDV) <65 mL (Group A) and normal heart size defined as LVEDV ≥65 mL (Group B) and associations between ECG outcome (false positive vs. no false positive) and heart size (small vs. normal) were analyzed using the Chi square test for independence, with a Yates continuity correction. LVEDV calculations were performed via a computer-processing algorithm. SPECT myocardial perfusion imaging was used as the gold standard for the presence of coronary artery disease (CAD).

**Results:**

Small heart size was found in 142 patients, 123 female and 19 male patients. There was a significant association between ECG outcome and heart size (χ^2^ = 4.7, p = 0.03), where smaller hearts were associated with a significantly greater number of false positives.

**Conclusions:**

This study suggests a possible explanation for the poor diagnostic accuracy of exercise stress testing, especially in women, as the overwhelming majority of patients with small heart size were women.

## Introduction

Cardiovascular disease is the leading cause of death in the United States claiming the lives of over 230,000 individuals each year [1]. Although the mortality from cardiovascular disease has recently been decreasing in men, it has been increasing for women [1]. Inaccurate diagnosis and ensuing management inefficiencies may contribute to the increased mortality [2].

Numerous studies have indicated that noninvasive cardiac stress tests have a lower diagnostic accuracy in women [2], [3]. In addition, diagnostic accuracy in women also varies depending on the test administered (i.e. stress echocardiography, stress myocardial perfusion imaging, pharmacologic or exercise electrocardiogram) [4]–[6]. Although sensitivity and specificity vary greatly between studies, as reported values depend widely upon patient selection criteria and methodological construct, studies using cross-gender comparisons consistently report lower diagnostic accuracy in female populations [4], [6]–[9]. The lower accuracy has been attributed to lower ECG voltage, smaller size of the coronary vessels, smaller heart size, hormonal factors (premenopausal relationship with endogenous estrogen levels), breast attenuation and functional impairment [2], [5], [8].

Specific to ECG diagnosis and ischemia, reports have indicated a higher number of false positive results in female patients when compared to male patients [6]–[8]. The purpose of this study was to evaluate and compare the diagnostic accuracy of the ECG during exercise stress test based on left ventricular (LV) cavity size.

## Methods

### Study Population

The study included 1,011 consecutive male and female patients who were referred for an exercise nuclear stress test at New York Methodist Hospital. All patients were referred for evaluation of chest pain (CP), dyspnea or other associated risk factors for CAD by their primary care physician or cardiologist. Patients with resting ECG's unsuitable for stress interpretation were excluded (pathologic Q waves, left bundle branch block (LBBB), left ventricular hypertrophy with strain pattern (LVH), Wolff-Parkinson-White (WPW) syndrome, or other significant (≥1 mm) downward displacement of the ST segment) [8]. Additionally, patients who did not reach a minimum of 85% predicted maximal heart rate during the exercise stress test were excluded from data analysis to standardize and ensure sufficient myocardial stress.

All patients underwent a thorough history and physical exam with data collected on presenting symptoms, past medical history, cardiac risk factors, as well as medications. The baseline ECG's were analyzed by a certified exercise physiologist prior to undergoing the stress test. All patients were instructed to hold their beta-blockers and calcium channel blockers for 24 hours prior to the stress test.

### Ethics statement

The study was approved by the Institutional Review Board (IRB) of New York Methodist hospital. An informed consent was not required because the study data was obtained and analyzed anonymously. The IRB of New York Methodist Hospital specifically waived the need for consent.

### SPECT ^201^Tl and Tc-99m Imaging

The gold standard for the diagnosis of ischemia was myocardial single photon emission computed tomography (SPECT) imaging. Initial resting myocardial SPECT images were acquired while the patient was in a supine position with shoulders flexed to 180°, using a GE Millennium MyoSIGHT rotating gamma camera (General Electric Company, Milwaukee, WI). Resting images were performed after receiving thallium (^201^Tl) (predetermined using a weight-based algorithm) for patients weighing less than 300 lbs using the following parameters: ^201^Tl dosing up to 4mcI; imaging time: 18 min; 36 views/ 30 sec per view; matrix 64×64; circular; Collimator: LEHR. Patients weighing greater than 300 lbs underwent resting images with technetium (Tc-99m), but followed the same parameters with the exception of a dosing rate of 12–15mcI.

Stress imaging for patients less than 300 lbs was performed with Tc-99m and used the following parameters: Tc-99m dosing range 30–40mcI; imaging time: 15 min; 36 views/25 sec per view; matrix 64×64; circular; 16 frames/RR interval. Again, the same parameters were used for patients weighing over 300 lbs, only the dosing ranged from 35–40mcI.

### Treadmill Exercise Testing

All treadmill testing was conducted using either the Bruce or Modified Bruce protocols (GE Medical Systems CASE Stress System Version 5 with Series 2000 Marquette Treadmill, General Electric Company, Milwaukee, WI). End points of exercise were predetermined according to absolute and relative indications for terminating exercise testing [12]. One minute prior to peak exercise, patients received Tc-99m (weight based) and were imaged 15 to 20 min post exercise. All 12 leads of the standard ECG were monitored and used for analysis. ST measurements were assessed visually 80 msec post J-point during exercise and recovery with the PR segments used as the baseline. The criteria for determining a positive exercise ST-segment response were as follows: ≥1.0 mm horizontal or downsloping depression 80 msec post J-point for at least 3 consecutive beats [12].

### Image Interpretation & Heart Size Criteria

The horizontal and vertical planes and the short-axis views were reviewed to detect the presence of defects. A 17-segment semi-quantitative method was utilized for visual interpretation of perfusion defects [13]. In addition, a semi-quantitative scoring system using the five-point model was utilized to assess myocardial perfusion (0–4: 0 = normal perfusion; 1 = mild reduction in counts; 2 = moderate reduction in counts; 3 = severe reduction in counts; 4 = absent uptake) [13]. An image was considered positive for ischemia if there was ≥1 segment perfusion defect seen at stress which was not seen at rest.

The left ventricular cavity size was determined by a computer algorithm that assesses the left ventricular cavitary borders and computes the size of the cavity in milliliters (mL). An end diastolic cavitary size less than 65 mL was used as a cutoff for small vs. normal cavitary size. This cut off was determined prior to initiation of the study by doing a retrospective analysis of data consisting of 200 consecutive patients to determine the smallest (20%) ventricular cavitary size in our patient population. The first 200 patients were performed on the same clinical grounds as the patients in our current study, using the new GE scanner and computer database system. This population was selected as it best estimated the population that we analyzed in the current study.

### Statistical Analysis

The diagnostic accuracy of the ECG with nuclear imaging as the reference standard was determined by calculating sensitivity, specificity, and predictive values [14]. Associations between demographic data and between ECG outcome (false positive versus no false positive) and heart size (small versus normal) were analyzed using the Chi square test for independence, with a Yates continuity correction. A multivariate analysis was performed to assess which pertinent variables (diabetes, age, body mass index (BMI), hypertension, coronary artery disease, and gender) were significantly associated with a false positive test. Both a C statistic and Goodness of fit were performed to assess the validity of the multivariate analysis.

Two-tailed significance was accepted at p<0.05 and all statistical analyses were conducted using SPSS® for Windows software, release 17.0 (SPSS Inc., Chicago, IL).

## Results

### Demographic data

A total of 1,011 consecutive patients were evaluated in the study sample and baseline characteristics provided in Table 1. Of the 1,011 patients, 482 were females, 359 had normal heart size and 123 had small heart size. There were 529 men, 510 with normal heart size and 19 with small heart size. The female patients accounted for the majority of small heart patients (86% vs. 13%).

**Table 1:** All patient characteristics (n = 1,011) including medications, symptoms and risk factors prior that completed the stress testing (treadmill) and categorized as small and normal heart size.

Small Heart Patients (n = 142)			Normal Heart Patients (n = 869)	
	*Total Number*	*% of Total*	*Total Number*	*% of Total*
***Medications:***				
ACE	42	29.6	254	29.2
B Blockers	36	25.4	255	29.3
Aspirin	46	32.4	309	35.6
Ca^2+^ Channel	18	12.7	102	11.7
Clopidogrel	10	7.0	88	10.1
Digoxin	0	0.0	3	0.3
Diuretics	25	17.6	106	12.2
Lipid Lower	49	34.5	305	35.1
Nitrates	4	2.8	37	4.3
***Risk Factors:***				
Hypertension	76	53.5	452	52.0
Dyslipidemia	87	61.3	463	53.3
Smoking	13	9.2	163	18.8
Diabetes	35	24.6	182	20.9
Stroke	3	2.1	14	1.6
Syncope	3	2.1	10	1.2
Lung disease	19	13.4	67	7.7
Renal disease	1	0.7	3	0.3
PVD	1	0.7	8	0.9
Age (years)	58.6±10.7		52.9±10.2	
BMI	28.9±5.8		30.5±6.6	
***Symptoms:***				
Angina	54	38.0	281	32.3
Arrhythmia	2	1.4	18	2.1
Chest pain	48	33.8	290	33.4
Dyspnea	43	30.3	243	28.0
Palpitations	35	24.6	231	26.6

ACE: angiotensin-converting enzyme inhibitors

B Blockers: Beta Blockers

Ca^2+^ Channel: calcium channel inhibitors

Lipid Lower: Lipid lowering agents

PVD: peripheral vascular disease

BMI: Body mass index

Patient's presenting complaints were most commonly chest pain (33.4%), angina (33.1%), dyspnea (28.3%) or palpitations (26.3%), although no differences were determined based on heart size (chest pain: χ^2^ = 0.01, p = 0.92; angina: χ^2^ = 1.8, p = 0.18; dyspnea: χ^2^ = 0.3, p = 0.57; palpitations: χ^2^ = 0.2, p = 0.63). Interestingly, there were a significantly higher percentage of smokers in the normal heart size patients (χ^2^ = 7.8, p<0.01). The most commonly prescribed medications throughout the sample population included ACE inhibitors (29.3%), lipid lowering agents (35.0%), aspirin (35.1%), beta-blockers (28.8%) and digoxin (<1%). Again, no significant difference in medications prescribed was observed between small heart size and normal heart size patients (ACE inhibitors: χ^2^ = 0.01, p = 0.93; lipid lowering agents: χ^2^ = 0.02, p = 0.89; aspirin: χ^2^ = 0.5, p = 0.46; beta-blockers: χ^2^ = 0.9, p = 0.33; digoxin: χ^2^ = 0.5, p = 0.48). Additionally, hypertension (52.2%), dyslipidemia (54.4%) and diabetes (21.5%) were the most commonly observed risk factors, and again, no difference was noted based on heart size (hypertension: χ^2^ = 0.1, p = 0.74; dyslipidemia: χ^2^ = 3.7, p = 0.06; diabetes: χ^2^ = 1.0, p = 0.32). Corresponding with the higher incidence of smokers in the normal heart size population, there was a significantly higher degree of lung disease in this group (χ^2^ = 5.0, p<0.05). The female patients with small heart size also were older (p<0.001, Table 1) and had a lower BMI (p<0.01, Table 1) than those women with normal heart size. During the treadmill stress test, an overwhelming majority of patients did not experience any chest pain or any abnormal blood pressure responses (Table 2).

**Table 2:** Stress test results for all patients and categorized under small and normal heart size.

	FEMALES			
	Small Heart (n = 123)		Normal Heart (n = 359)	
*Stress Variables:*	*Total Number*	*% of Total*	*Total Number*	*% of Total*
Normal BP response	114	92.7	321	89.4
No CP	115	93.5	319	88.9
CP (atypical)	2	1.6	4	1.1
CP (typical)	3	2.4	12	3.3
CP (unspecified)	3	2.4	24	6.7

	MALES			
	Small Heart (n = 19)		Normal Heart (n = 510)	
*Stress Variables:*	*Total Number*	*% of Total*	*Total Number*	*% of Total*
Normal BP response	16	84.2	442	86.7
No CP	18	94.7	444	87.1
CP (atypical)	0	0.0	9	1.8
CP (typical)	0	0.0	26	5.1
CP (unspecified)	1	5.2	31	6.1

BP: blood pressure.

CP: chest pain.

### ECG diagnostic accuracy

All false positive ECG results for small heart size patients (n = 31) and normal heart size patients (n = 91) and all false negative results for small heart size (n = 13) and normal heart size (n = 146) are depicted graphically in Figure S1. Twenty-nine women with small heart size were determined to have false positive ECG, 12 false negative, 16 true positive and 66 true negative interpretations. Forty-one women with normal heart size were determined to have false positive ECG, 42 false negative, 66 true positive and 210 true negative interpretations. There was a significant association between ECG outcome and heart size (χ^2^ = 4.7, p = 0.03), where smaller hearts were associated with a significantly greater number of false positives. On multivariate analysis, diabetes was found to be significantly associated with a false positive test (p = 0.03). Both age and heart size had a trend towards significance with p = 0.05 and p = 0.08, respectively. The goodness of fit (P = 0.08) indicated very poor accuracy of this multivariate analysis and the C statistic (P = 0.65) indicating only moderate precision.

Within the male patients, 19 were categorized with small hearts. Only two of the 19 patients demonstrated false positive ECG readings, while only one was false negative, three were true positive and the remaining 13 were true negative for ischemia. For the 510 males categorized as normal hearts, 50 were false positive, 104 false negative, 96 true positive and 260 true negative. The sensitivity, specificity and predictive values of exercise ECG in the total study population, female patients and male patients are demonstrated in Table 3.

**Table 3:** Comparison of ECG diagnostic accuracy of: a) all exercise stress tests; b) all female patients (n = 482); c) male patients (n = 529).

*All Subjects*		
Treadmill	Small Heart (n = 142)	Normal Heart (n = 869)
Sensitivity	59.4%	52.6%
Specificity	71.8%	83.8%
Positive Predictive Value	38.0%	64.3%
Negative Predictive Value	85.9%	76.3%

*Females*		
Treadmill	Small Heart (n = 123)	Normal Heart (n = 359)
Sensitivity	55.6%	61.8%
Specificity	69.2%*	82.2%
Positive Predictive Value	34.9%*	58.5%
Negative Predictive Value	84.0%	84.1%

*Males*		
Treadmill	Small Heart (n = 19)	Normal Heart (n = 510)
Sensitivity	75.0%**	48.0%
Specificity	86.7%**	83.9%
Positive Predictive Value	60%**	65.8%
Negative Predictive Value	92.9%**	71.4%

* = significantly different from normal heart size (p<0.001).

** = unable to conduct statistical analysis due to insufficient sample size.

## Discussion

The unique finding of this study was that patients with smaller LV chamber sizes were more likely to display a false positive ECG result during a maximal exercise stress test than patients with a normal chamber size. The overwhelming majority of patients with small heart size were women.

### Possible mechanisms

Several studies have addressed the lack of diagnostic accuracy in ECG stress testing in women [7], [8], [15], [16]. Sensitivity and specificity results from these studies range from 31–80% and 41–68%, respectively [17], [18]. The current hypothesis' in the literature for the lower diagnostic accuracy in women include: breast attenuation, coronary vessel size, hormonal factors (pre-menopausal estrogen or post menopausal hormone replacement therapy), and/or a lowered exercise tolerance/capacity [7], [8], [15], [16]. One anatomical mechanism that has not been addressed in detail, however, is the physical dimension of chamber size and the potential relationship to stress ECG tracings. In the current study, small chamber size (LVEDV<65 mL) was indicative of higher rates of false positive ECG diagnosis in women.

To our knowledge, only Hansen and colleagues have addressed diastolic cavitary size and the relationship between nuclear stress testing [5]. Others have found correlations between large body size and reduced cardiac workload [19]. However, this would not explain the high rate of false positives observed in the current study as all of the patients included in the analysis, regardless of heart size, attained a high workload (as indicated by 85% of predicted HRmax).

There are other possible explanations as to why left ventricular chamber size may affect the diagnostic accuracy of the exercise ECG. Small heart size may have associated smaller coronary arteries leading to diffuse subendocardial balanced ischemia which may not be picked up on nuclear SPECT imaging. This would imply that the gold standard is inaccurate and not the exercise stress ECG itself. A third possible explanation may be that patients with a small heart size need to have a different lead placement than patients with normal heart size as perhaps the position of the leads relative to the myocardium can affect the ST segment. In addition, small heart size changes the dynamics of the relationship between the R wave and the ST segment which could potentially be another mechanism for the increase rate of false positive ECG changes in patients with small heart size.

In our study, we used automated gated SPECT for the quantification of left ventricular (LV) volumes. Gated SPECT has become the state-of-the-art technique for myocardial perfusion imaging and it combines the evaluation of both myocardial perfusion and function in one setting. Studies conducted on a large cohort of patients have not only demonstrated a high reproducibility of LV volume quantification by automated gated SPECT, but also a good correlation with traditional techniques like echocardiography, contrast angiography or pooled blood studies [20]–[24]. For example, in a study conducted by Maruyama et al, the LV volumes and LV mass index calculated by QGS SPECT were consistent with those quantified by M-mode echocardiography, by Devereux's method [25]. Even though few smaller studies looking at the older gated SPECT in patients with small hearts postulated that LV volumes can be over-estimated due to limited spatial resolution, larger studies using appropriate image acquisition parameters and quality control methods validated gated SPECT in such patients [26], [27]. In addition, the newer and commonly used software for Cedar-Sinai QGS with a SPET filter, high cut-off frequency and appropriate zooming, and Emory tool box or layer of maximum count method are well-correlated [28]–[31]. Furthermore, in studies done by Gimelli et al, the authors demonstrated a superior predictive value of gated SPECT derived volumes compared to those derived from echocardiogram [32], [33].

### Potential limitations

A potential limitation of this study was using nuclear imaging as the criterion measure and not coronary angiography. However, other studies have justified the accuracy and predictive capacity of nuclear SPECT imaging and using myocardial perfusion imaging rather than anatomical angiography may be more relevant in determining ischemia [10], [11]. Hansen et al., described a lower diagnostic accuracy of SPECT imaging based on small heart size [5]. However, using coronary angiography as a gold standard has its own problems as one is then using an anatomical imaging modality rather than a functional imaging modality. The range of angiographic stenosis, which results in ischemia, is quite broad and dependent not just on the severity of a stenosis but also on the length of stenosis and the degree of atherosclerotic disease proximal and distal (e.g. 2 tandem 50% stenosis may be more hemodynamically significant than one 70% stenosis). In addition, determining the presence of CAD based on a secondary or tertiary branch with significant stenosis can also lead to problems in determining the accuracy of a diagnostic test for the presence of ischemia. Ideally, obtaining fractional flow reserve on all patients referred for angiography would help alleviate many of these problems; however, this is quite burdensome for both the patient and the catheterization laboratory.

Even though it is ideal to correlate the stress ECG and nuclear stress results with coronary angiographic data, we were interested in investigating the functional strategy for the diagnosis of myocardial perfusion using non-invasive modalities, instead of anatomic evaluation. In addition, we are unable to retrospectively review this data as our archive for angiography during this time period has been corrupted and the data is lost.

It is possible that a larger body mass index would be more prone to attenuation artifacts due to either breast attenuation or other soft tissue attenuation, which would lead to more positive test results in the gold standard. Outcome data in future studies would be required to better assess the contribution of larger body mass index on the accuracy of SPECT imaging.

Another potential limitation is that hormonal status i.e. post menopausal (in the female population) were not accounted for in the patient population.

### Clinical implications

It is well-known that plain exercise-ECG test has high false positivity. Our study strongly suggests that certain groups of population like older women and particularly women with lower BMI who have smaller body size may have smaller heart and probably smaller coronary arteries. Patients with these characteristics on physical examination may probably benefit more being referred to imaging stress tests when indicated, compared to the conventional exercise ECG stress test because of high false positive results. In addition, patients with small left ventricular volumes and mass by echocardiography also should be considered for stress imaging instead of exercise ECG.

### Conclusions

The exercise ECG has a higher false positive rate in patients with small heart size when compared to patients with normal heart size. As the overwhelming majority of patients with small heart size were women, the results offer additional insight into a potential explanation for the poor diagnostic accuracy of stress testing in women.

## Supporting Information

Figure S1 False Positive and False Negative comparison for (a) Female patients; (b) Male patients; (c) all patients.
(DOC)
