# Supplementary material for: The Accuracy of the Electrocardiogram during Exercise Stress Test Based on Heart Size
Source: PLoS One. 2011 Aug 17;6(8):e23044. doi: 10.1371/journal.pone.0023044 (PMC3157363; doi:10.1371/journal.pone.0023044)
Supplement: Figure S1 — False Positive and False Negative comparison for (a) Female patients; (b) Male patients; (c) all patients. (DOC) [file pone.0023044.s001.doc]

**Figure S1:** False Positive and False Negative comparison for (a) Female patients; (b) Male patients; (c) all patients.

1. *Female Patients*

n=12

n=41

n=42

n=29

1. *Male Patients*

n=2

n=1

n=50

n=104

*c) All Patients*

n=31

n=13

n=91

n=146
